# Value misalignment in X’s feed algorithm is a reflection of value tensions in engagement

**DOI:** 10.1073/pnas.2610388123

**Published:** 2026-08-17

**Authors:** Ziv Epstein, Farnaz Jahanbakhsh, Tiziano Piccardi, Axel Peytavin, Isabel Gallegos, Shardul Sapkota, Dora Zhao, Johan Ugander, Michael S. Bernstein

**Affiliations:** ^a^ Schwarzman College of Computing, Massachusetts Institute of Technology, Cambridge, MA 02143; ^b^ Electrical Engineering and Computer Science, University of Michigan, Ann Arbor, MI 48109; ^c^ Computer Science Department Johns Hopkins University, Baltimore, MD 21218; ^d^ Computer Science Department Stanford University, Stanford, CA 94305; ^e^ Department of Statistics & Data Science Yale University, New Haven, CT 06511

**Keywords:** social media, algorithmic personalization, media amplification, value alignment

## Abstract

Social media feed algorithms rank content that is purported to be preferred by users, but the engagement behaviors that drive these algorithms are (at best) indirect proxies for users’ explicitly self-stated values. Are the resulting feeds value aligned, and if not, why? We investigate this question by annotating the basic human values expressed in participants’ X (Twitter) feeds (N = 715 US users), analyzing the relationship between the posts’ value expressions and the posts’ amplification in the ranked “For You” Page feed, and then comparing the amplified values to users’ own values. We observe that the inventory of posts from followed accounts reflects users’ self-stated values—but that there is an overall negative correlation (misalignment) between users’ explicit values and the value expressions the algorithm is more likely to amplify. We turn to engagement behavior to understand this misalignment and observe that users’ engagement behaviors can be misaligned with their stated values—likely causing the algorithm to learn and reflect these misaligned values. We also detect partisan differences consistent with this theory: While the algorithm amplifies values negatively correlated with both Democrats’ and Republicans’ self-stated values, they are more misaligned for Democrats. And in fact replying, a heavily weighted form of engagement, is associated with values that are less aligned for both Democrats’ and Republicans’ self-stated values, and is even more misaligned for Democrats. Taken together, these findings offer a glimpse into the tensions between the values that people hold and those that provoke reactions, and how these value tensions can produce misaligned outcomes.

Algorithms that rank social media feeds take into consideration a set of posts including those from accounts that an individual follows—their in-network inventory—and make a personalized selection from this inventory. These algorithms are typically optimized for engagement, promoting content to maximize an objective that weighs the user’s predicted interactions (1, 2). Toward the platforms’ high-level goal of user retention, this design is intended to promote content that users value by implicitly learning their preferences from engagement behavior (3).

Yet concerns over the spread of misinformation, promotion of toxic content, and polarized ‘filter bubbles’ have raised questions about the perverse effects of engagement optimization (4). The field of value alignment has also revealed the broader concern that algorithms can systematically fail to encode people’s explicit values, instead mirroring reactive behaviors that depart from such values (5).

How does this “value duality” of inferred preferences from engagement prediction on one hand and engagement patterns that diverge from explicit values on the other intersect to shape the overall values amplified by these algorithms? Do we ultimately create algorithms that reflect self-stated values? Or does our confrontation (6) and animosity-driven engagement with content that diverges from these values (7–9) produce fundamental algorithmic misalignment? In this paper, we explore “the value alignment” of social media algorithms by analyzing the association between user’s values and those the algorithm promotes. In this work, we use the lens of basic human values to reveal the gap between users’ self-stated values and those that provoke reactions (8), and how these value tensions shape engagement-based ranking algorithms. To do so, we draw on Schwartz’s refined theory of Basic Human Values (10), a set of 19 values that nest into a three-level hierarchy and has been validated across cultures (Fig. 1). We use a validated method for annotating expressions of Schwartz values in X posts (11) (*SI Appendix*, section 2) to measure the values associated with algorithmic promotion from a user’s inventory to their “For You” feed (FYP), and compare this value amplification with user engagement patterns.

**Fig. 1.**
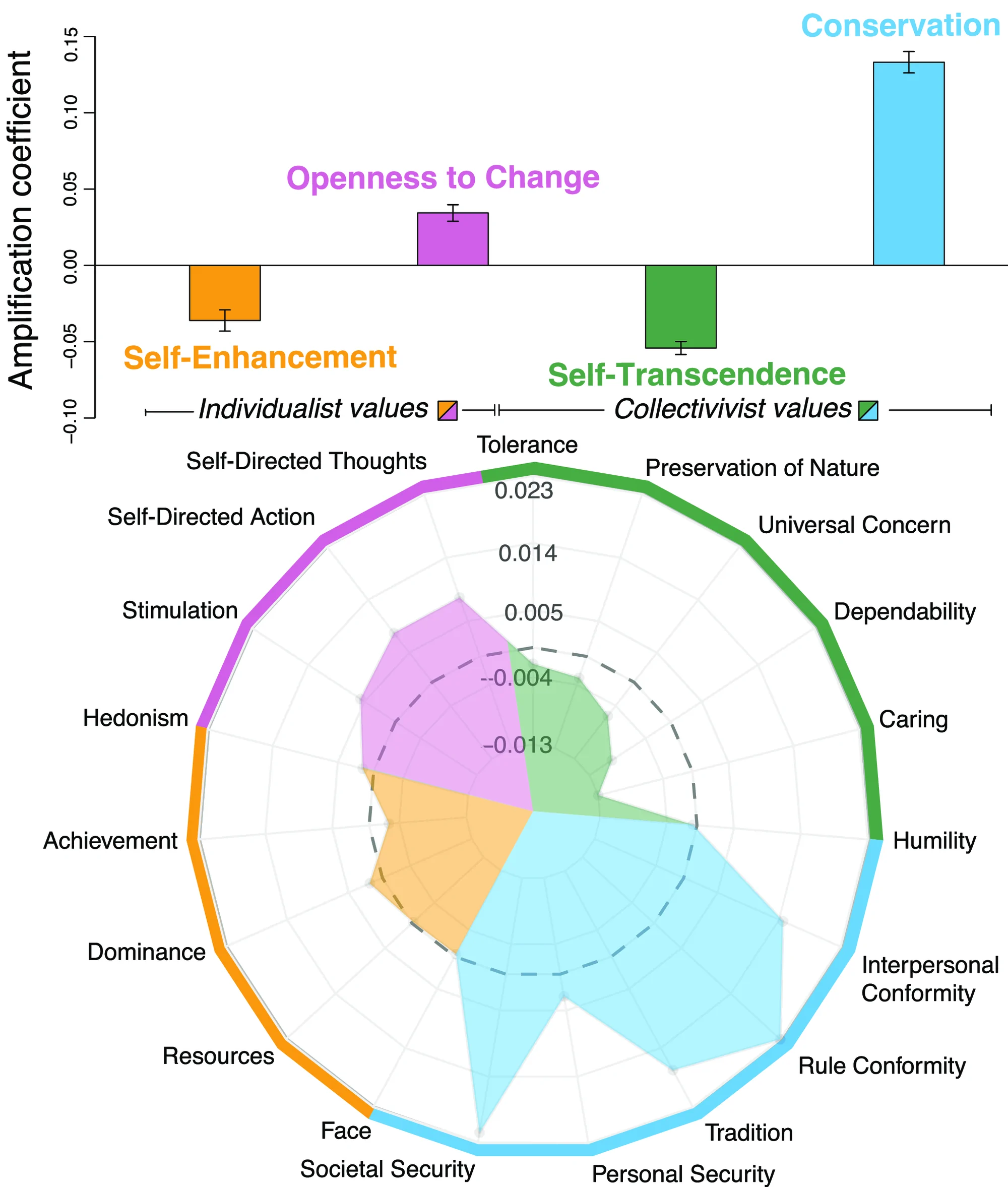
*Top*: Amplification results for the four middle-level value clusters of *Self-transcendence*, *Openness to change*, *Conservation*, and *Self-enhancement*. *Bottom*: A radar plot of amplification results for low-level values using coefficients computed from the preregistered PCA analysis (*Materials and Methods*).

We recruited a nationally representative sample of N=715 Americans who are active users of X in September and October 2024, quota matched on ethnicity, gender, and partisanship, to install a browser extension to collect their FYP and Following feeds (i.e. the in-network inventory, see *SI Appendix*, section 1). We measure value amplification by regressing amplification on the values expressed in a post over the in-network inventory (*Materials and Methods* and *SI Appendix*, section 3) and compare these results to the users’ own engagement within 24 h after feed collection.

## Results

First, we identify which values are amplified by the FYP algorithm. At the highest level of the Schwartz values hierarchy, we observe more amplification of individualist values (β=0.023) than collectivist values (β=0.002,P<0.001). However, at the intermediate level that considers the four clusters that comprise individualism and collectivism (*Self-transcendence*, *Openness to change*, *Conservation*, *Self-enhancement*, see Fig. 1, *Top*), we see heterogeneity: On one hand, the individualistic cluster of *Openness to change* is amplified (β=0.034,P<0.001) while the individualistic cluster of *Self-enhancement* is demoted (deamplified, β=−0.036,P<0.001). And on the other hand, the collectivist cluster of *Conservation* is amplified (β=0.133,P<0.001) while the collectivist cluster of *Self-transcendence* is demoted (β=−0.0541,P<0.001). For low-level values (Fig. 1, *Bottom*), we observe the highest rates of amplification for conservation values such as rule conformity (β=0.023), societal security (β=0.021), and tradition (β=0.017). We also observe demotion of self-transcendent values such as caring (β=−0.012), dependability (β=−0.009), as well as universal concern (β=−0.006), and preservation of nature (β=−0.003).

**Fig. 2.**
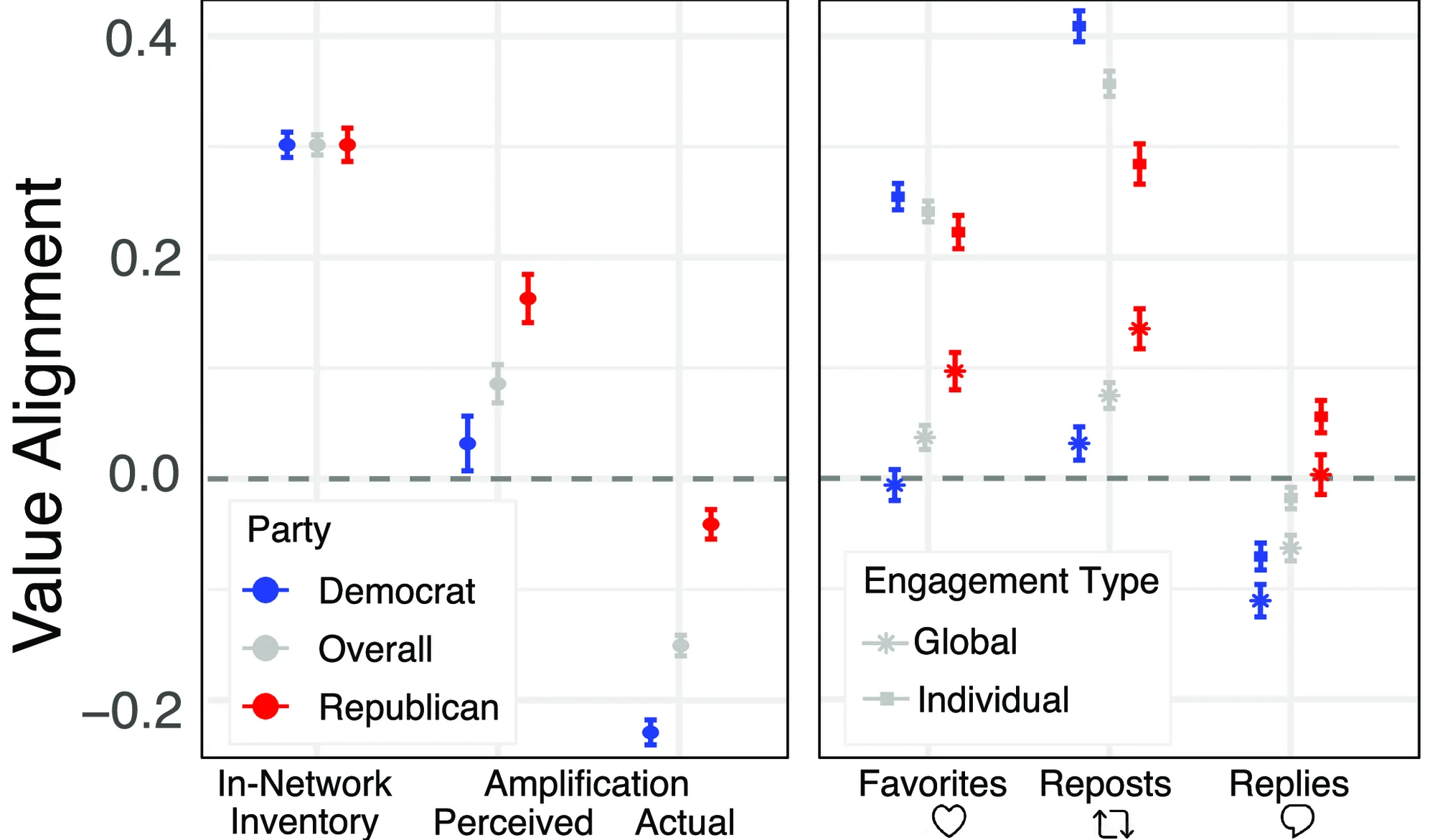
Value alignment (Spearman rank correlation with personal values) for Democrats (Blue), Republicans (Red), and Overall (Gray). (*Left*) Value alignment for in-network inventory, amplification (Fig. 1) and perceived amplification. (*Right*) Value alignment for participant’s own engagement patterns up to 24 h after the study (square) and overall “global” amount of engagement that posts accrued (star).

But *whose* values are amplified? In Fig. 2, *Left*, we compare individuals’ self-stated values to both the values expressed in their social media inventory and the values amplified by their feed ranking algorithms. We compute the mean Spearman rank correlation between each individual’s personal values and the frequency of values in user’s in-network inventory, the amplification coefficient for each value, and the value-level coefficients estimated for engagement (*SI Appendix*, section 4). We observe that individuals’ values are broadly aligned with the values represented in their inventories ($\overset{¯}{\rho }=0.302$, 95% CI=[0.284,0.320], meaning that people follow accounts that generally produce content aligned with their values. This does not differ across political party (*t* test, t=0.001, and P=0.999).

However, beyond this alignment between self-stated values and inventory, we observe participants’ values to be negatively correlated with the likelihood of content amplification ($\overset{¯}{\rho }=-0.150$, 95% CI=[−0.169,−0.132]), suggesting the algorithm is actually amplifying content that is *misaligned* with users’ explicit values. Here we observe heterogeneity by political identity: Democrats’ amplification ($\overset{¯}{\rho }=-0.229$) is less aligned with their values than Republicans’ ($\overset{¯}{\rho }=-0.041$ and P<0.001).

We find that the values that people perceive are amplified by the algorithm follow the same partisan spread pattern as actual amplification, with Democrats perceiving the algorithm as less aligned with their values than Republicans (${\overset{¯}{\rho }}_{\text{Rep}}=0.162,{\overset{¯}{\rho }}_{\text{Dem}}=0.031$, $p_{\text{diff}}<0.001$), but the extent of the actual misalignment is significantly greater than people think.

As a robustness check, we conduct a preregistered replication study that largely finds the same pattern of results (*SI Appendix*, section 6). While the replication also found significant partisan differences in alignment between users and amplification (P<0.001), we found a small but positive alignment for Republicans (ρ=0.0372) and a negative alignment for Democrats (ρ=−0.199), pointing to the stronger claim that X’s algorithm could be aligned for Republicans but misaligned for Democrats (*SI Appendix*, section 6).

### Explaining the Partisan Value Gap.

Why is amplification value-*misaligned* for users, despite the fact that users’ inventories are aligned with their explicit values? Since the algorithm learns from engagement behavior, we leverage two datasets of engagement behavior to explain this “value gap”: 1) participant’s engagement behavior during 24 h after they installed the Chrome extension (personal), and 2) the overall engagement metrics of posts in participants’ feed (global). For individual favoriting and reposting, we find high levels of value-alignment for both Democrats (${\overset{¯}{\rho }}_{\text{Fav}}=0.254,{\overset{¯}{\rho }}_{\text{Repost}}=0.408$) and Republicans (${\overset{¯}{\rho }}_{\text{Fav}}=0.222,{\overset{¯}{\rho }}_{\text{Repost}}=0.284$), Fig. 2, *Right*. However, we also find evidence suggesting that replying is less aligned with explicit values than these other more intentionally curatorial forms of engagement, and are in fact even value-misaligned on average for Democrats (${\overset{¯}{\rho }}_{\text{Dem}}=-0.0702,{\overset{¯}{\rho }}_{\text{Rep}}=0.056$). This corroborates work showing replies can act as value-misaligned confrontations that drive engagement (6). Despite the fact that replying is a sparse event (only 6.8% of engagement events), it is known that X weights predicted replies more heavily than predicted favorites [up to 27× more (12)]. Thus, it is possible that Democrats reply to value-misaligned content which steers the algorithm to differentially amplify value-misaligned content for them. Such a mechanism has the potential to create a misaligned feedback loop, a flywheel where value-misaligned content is further shown to those users, who reply to it, thus amplifying more such content for more replies.

We find that the values associated with *global* engagement on the platform (number of favorites, retweets, replies) are not only less value-aligned than the values of posts individuals engage with *personally* (for both retweets and favorites), but also that they are systematically more aligned with Republican users than Democrat users (Fig. 2*B*, star). On one hand, this suggests that global engagement on the platform (which is principally constituted by posts espousing Republican values) preferentially aligns with Republican users, shaping both users and the algorithm’s inputs. On the other, it suggests that Republican users encounter and reply to content that is more aligned with their values, mitigating for them the value-misaligned flywheel that Democrat users are particularly susceptible to.

### Discussion.

Methodologically, this paper offers an opening for understanding and evaluating social media ranking algorithms without internal access to the platforms, connecting in-network amplification with constructs of societal relevance. Substantively, we find that, on average, X’s feed algorithm amplifies engaging content, but ultimately, this content is misaligned with many users’ own values, particularly Democrats’. This general pattern aligns with past work identifying amplification of conservative content (4, 13).

But we also reveal that this partisan “value gap” could be explained by an asymmetric flywheel whereby Democrat users confront the abundant value-misaligned content by replying to it, which the algorithm in turn preferentially learns from and continues to feed them. This highlights a core tension with how engagement-maximizing algorithms operate on social media: Frictions between users’ stated preferences and their behaviors of reactive confrontation are exploited by engagement-maximizing algorithms to create runaway feedback loops of increasing value misalignment. We note that while our findings are consistent with the theoretical account of a confrontation effect, future work must test this proposed mechanism account causally and at high power.

These findings raise important questions for the design of social media algorithms, particularly in polarized contexts that skew along partisan lines: What are the effects of asymmetric alignment in amplified values? What forms of engagement and attention should these algorithms learn from? Whose values should these algorithms align with, system designers, the “average” user, or each user individually? The answers to these questions require new and distinct paradigms for content moderation, regulation, and user control.

## Materials and Methods

This work has been approved by Stanford IRB protocol #IRB-68917. Informed consent was obtained from all participants.

To measure basic human values, we annotate values in posts using an LLM fine-tuned on human annotation and a personalization architecture to account for interrater annotation disagreements (11) (*SI Appendix*, section 2). To measure amplification, our primary preregistered regression models use as independent variables the first four principal components of a PCA for the 19 low-level values (these four principal components explain 90.37% of variance across these values) and random effects for users (*SI Appendix*, section 3). We collected participants’ individual engagement events (likes, reposts, and replies) from the browser plugin for 24 h after feed collection, corresponding to 31,536 view events from N=1,023 participants and 961 unique engagement events from 149 participants that have labeled value labels. We also predict engagement from the values expressed in viewed posts (*SI Appendix*, section 5A). We also collect the engagement metrics (number of likes, reposts, and replies) from posts from participant’s in-network inventory and normalize these counts by log transforming them and predict engagement as a function of the Schwartz values expressed from inventory posts (*SI Appendix*, section 5B).

## Supplementary Material

Appendix 01 (PDF)

## Data Availability

Anonymized study data and anonymized survey data have been deposited in GitHub (https://github.com/zivepstein/pnas-feed-values) (14).
