# Supplementary material for: Value misalignment in X’s feed algorithm is a reflection of value tensions in engagement
Source: Proc Natl Acad Sci U S A. 2026 Aug 17;123(34):e2610388123. doi: 10.1073/pnas.2610388123 (PMC13506001; doi:10.1073/pnas.2610388123)
Supplement: Supplementary file 1 [file pnas.2610388123.sapp.pdf]

Supplementary Information for

Value misalignment in X's feed algorithm is a reflection of value tensions in engagement

Ziv Epstein, Farnaz Jahanbakhsh, Tiziano Piccardi, Axel Peytavin, Isabel Gallegos, Shardul Sapkota, Dora Zhao, Johan Ugander, and Michael Bernstein

Corresponding authors: Ziv Epstein and Michael Bernstein.
E-mail: zive@mit.edu, msb@stanford.edu

This PDF file includes:

Supplementary text
SI References

Supporting Information Text

1. Inventory Collection

We recruited 1000 participants via Prolific, to use our Chrome Extension in September and October 2024, across 3 waves of data collection (Sep 23-29, Oct 18 and Oct 29-Oct 31). Of those 1000, 715 made it to the post-data collection survey and we identified a match between their survey data and extension data. Participants were all from the United States and were required to be 18 years old or older. We recruited a nationally representative sample of social media users quota matched on ethnicity, gender, partisanship and age. However, we were unable to meet the nationally representative quota for participants in the age range 55-100 due to the fact that we required participants to have an active social media (X) account to participate. Nonetheless, 7.3% of our sample was over the age of 55. Within this sample, the median age was 35 (min=18, max=95[*]), 72.58% self-reported White/Caucasian, 43.3% female and 34.2 %Democrat, 26.7% Republican and 33.4% Independent.

Participants were asked to install a Chrome Extension that loaded their X homepages and then used it to download posts from both their For You (FYP) and their Following feeds simultaneously. The Chrome Extension aims to collect 75 batches of tweets (groups of tweets that load together) from the FYP in addition to as many Following Batches as necessary until it encounters a tweet posted more than 7 days prior to the time of retrieval or as many as possible within 30 minutes, whichever happens sooner.

The rationale for the number and criteria for feed collection is as follows: Heavy Ranker—the recommender system used to rank the tweets in FYP—is fed as input approximately 1500 tweets that are selected from a pool of recent in-network and out-of-network tweets (1)[†]. Then, at the last stage of the pipeline, promotions including ads and recommendations about who to follow are additionally mixed into the ordered set. With the conservative assumption that within each FYP batch there exist 20 non-promotional tweets, fetching 75 batches ensures that we have collected all the tweets output from one run of the Heavy Ranker. Our focus is on investigating which in-network tweets are amplified in the FYP. Because the Heavy Ranker only processes "recent" tweets, we also limit the Following feed collection to recent tweets. While the exact definition of "recent" is unclear, our pilot tests suggest that tweets older than 7 days do not appear in the FYP. Thus, we constrain the Following feed collection to tweets posted within 7 days prior to retrieval. We filter to participants for whom we identify at least 30 tweets that are algorithmically amplified from their in-network inventory to their For You page.

Given that we collect longer time frames of Following posts than For You posts, we limit our analysis only to a timeframe where we would expect amplification to occur. In particular, for a given user, we limit to all Following posts that appear in 6-hour time blocks where we observed nonzero amplification i.e. at least one post that appears in their For You feed, resulting in a dataset of 2,952,899 inventory posts across the 715 participants. To capture context, the text of a given tweet (tweet_1) made in reply to or quoting another tweet (tweet_2) is represented as "tweet_1 REPLY TO: tweet_1" and "tweet_1 QUOTED: tweet_1" respectively.

Given this dataset of 2.9M posts, with an average of 4,129.9 posts from the Following page per user, of which on average 524.3 were amplified into the For You page, we construct a binary indicator of whether or not each post in a given user's Following page appeared in the algorithmically ranked FYP page (`is_amplified`), with an overall average amplification rate of 0.127.

2. LLM model for value annotation

Once the inventories have been collected of our participants, we must then annotate the posts in these inventories for values. To do so, we use the method developed and explained in (3). For convenience, we elaborate on the core components of this approach below.

A. Sourcing a Dataset of Social Media Posts. First, we recruited a sample of social media users (N=281) quota-matched to be a representative sample of the US population on age, ethnicity, gender and partisanship using Prolific in May 2024. Participants were all from the United States and were required to be 18 years old or older. We were unable to meet the quota for participants in the age range 55-100 (92/109) so we upsample posts from participants in this age range by 18.4% when constructing our silverset of social media posts. Within this sample, the median age was 43 (min=19, max=80), 58.36% White/Caucasian, 51.2% female and 30.6% Democrat, 28.8% Republican and 40.6% Independent. Participants were paid ∼ $15.42 an hour and took approximately 7 minutes to complete the study.

After they consented to the research, participants were asked to install a Chrome Extension that downloaded posts from their X (Twitter) feeds, both their algorithmically curated For You page (FYP) and their in-network Following feeds simultaneously. We collect and annotate posts from both the Following feed and FYP to account for any systematic differences that may exist between them.

When a browser opens the X page, tweets are fed to the browser client in "batches": a single API request returns a set of tweets at a time. The Chrome Extension aims to download 16 batches of tweets: 7 from the FYP, and 9 from the Following page. Each batch for FYP consists of approximately 30 tweets, whereas the Following feed returns a variable number of tweets up to 100.

[*] one participant who was over 55 and had 17 years of education accidentally typed "5" into the continuous age question, so we omitted this observably incorrect Age number from demographic summaries. Therefore the age demographics reported here represent *a lower bound* on the true age our participants.

[†] At the time of our study, approximately half of content in a X/Twitter user's "For You" feed was in-network (inventory) content, and the other half out-of-network content (2).

From the 281 participants, we collected 265,442 posts, with an average of 207 posts from the FYP and 776 from the Following page per user. Among these 265,442 posts, there are 151,740 posts with unique, non-empty text in English (using Google's Chromium language detection tool `cld2`). We also screen out private posts (n=6,025; 3.97%), deleted posts (n=2,004; 1.32%) and/or reply tweets that are replying to posts not displayed in the user's feed (n=7,581; 4.99%) to focus only on the 142,652 public posts we can render with proper context.

We next prepare these posts for human annotation, since some posts are either inappropriate (NSFW) to show to annotators or incomprehensible to most annotators. We filter the posts for comprehensibility (grounded in the constructs of readability, coherence, spam behavior, and context required for understanding) and NSFW (to comply with our institution's IRB for data annotation) using an automated GPT-based pipeline, with the prompt that rates all 19 values jointly each on a 0-6 scale based on a short description with `GPT-4o`, temperature=1.0 and seed=0. We filter out any posts that score less than 3 on a comprehensibility scale, resulting in a dataset of 89,383 (62.6%) posts. We then filter out 1,937 posts with NSFW content, resulting in a final dataset of 87,446 posts.

The occurrence rate of different values in social media posts varies, as do the numbers of posts per person across both Following and FYP feeds. Therefore, uniform sampling procedure may yield a sample that underrepresents rarer values and overrepresents certain users for annotation. To construct a final set of posts for human annotation, we stratify on user and post source as well as on a preliminary LLM-based measure of value expression tuned for recall over precision to prioritize catching as many potential value-expressing posts as possible. Since stratification requires an initial estimate of which value a post may express, we first built an automated classifier tuned for recall rather than precision—that is, we prioritized sampling as many potential value-expressing posts as possible, even if this meant including some false positives.

In particular, we perform a preliminary rough classification of these posts for all 19 of the Basic Human Values (4) on a 7-point Likert scale [0-6] using another automated GPT-based pipeline with `GPT-4o`, temperature=1.0 and seed=0. This allows us to upsample posts that may reflect rarer values. For this upsampling, we say a given post reflects a given value if the GPT annotation pipeline scored it a 4 or above. We then sample a final set of 5,227 posts for human annotation by stratifying across users, post source (FYP vs following), and value expression. In particular, for each user and each value, we sample a tweet from that user's feed that reflects that value. We sample FYP and Following posts with equal probability, but for a given user, if only one type of post exists, we return that tweet. Because we did not meet our quota for participants over the age of 55 (by 17 participants), we randomly sample (without replacement) 17 of the 76 participants over the age of 55 who completed the survey and repeat this process for each of them to ensure representation of posts from users over the age of 55. Of the 5,227, 3,182 came from users' Following feeds (60.8%) and 2,045 (39.1%) came from users' FYP.

This process yielded a dataset of 5,227 posts that we can use to study human value annotation and to predict the values expressed in those posts. In this section, we describe our ground truth data annotation method.

B. Annotating the Dataset of Social Media Posts. In total, 1,276 participants began the survey. We filtered out N=153 participants who failed two simple attention checks (see Appendix) or who did not finish the study. N=1,123 completed the study. Our final dataset included ratings from N=1,079 participants who have a full set of ratings for all 30 posts assigned to them and within this sample, the median age was 45 (min=18, max=85), 61.2% White/Caucasian, 50.8% female and 29.6% of participants lean Democrat vs 28.63% lean Republican. Participants were paid $15.00 an hour and took approximately 60 minutes to complete the study.

Each participant provided value expressions ratings for 30 posts. The posts for each participant were randomly sampled from the dataset of 5,227 posts sourced as described above (each post was rated by a median of 6 people). Given this sampling procedure, we retained ratings of 5,211 posts (since posts are sampled with replacement for each participant).

We used a recursive labeling where raters could traverse the value tree to elicit value ratings for all 19 values in an efficient manner. The branching method leverages the tree structure of the Schwartz Theory of Basic Values , starting with the high-level values, and traversing down only those branches whose parent values they had marked as expressed in the post.

Following this primary task, participants completed the Value Calibration Questionnaire (VCQ) of 25 additional ratings on a shared set of questions. Finally, participants answered a number of demographic and political questions.

To familiarize them with the task and to determine eligibility for proceeding to the main labeling task, participants first completed a short training for four synthetic posts followed by a gating task. The training introduced annotators to the meaning of the Schwartz values, while the gating task assessed their understanding. These training posts were created to unambiguously reflect particular values, and participants were not allowed to proceed until they checked the right answer. If they answered incorrectly, a message appeared giving them the correct answer and asking them to try again.

After training, they proceeded to the gating phase, where they rated four posts that, based on a pre-study, either unambiguously contained or did not contain the values of self-direction action or face. Participants who got one or fewer of the four correct were not allowed to continue. We took care in selecting the questions for both pre-tasks and in setting the eligibility threshold for the gating task to ensure a shared baseline understanding.

After completing basic attention screeners and training, participants who successfully passed the gating phase proceeded to rate 30 posts drawn at random from the larger set of 5,227 posts for value expression using the branching method discussed above. When presenting a tweet to our annotators for value annotation, to convey context, the text of a given tweet (tweet_1) made in reply to or quoting another tweet (tweet_2) was represented as "tweet_1 REPLY TO: tweet_1" and "tweet_1 QUOTED: tweet_1" respectively. The annotation dataset we create is available for researchers on a case by case basis to preserve the privacy of the participants. It is stored securely in an de-identified manner.

C. Training and evaluating the model. Due to the subjective nature of values (3), we use a personalized model which predicts *the extent to which an individual perceives* a value in a social media post. This model uses post-level and individual-level features to predict value expression. The post-level features are the consensus predictions from a gpt-4o model fine-tuned on consensus annotations. The individual-level features are that individual's responses to the 25 VCQ questions. For each post–user pair, these models predict that user's 0–6 rating for a given value V. We train 19 random forest models (one per value) trained on the ratings of 3000 posts stratified by number of ratings (see (3) for details). This model has been shown to outperform both off-the-shelf LLM methods and fine-tuned methods (3). We use these random forest models to annotate the inventory posts for values.

3. Amplification Analysis

The Following feed is a reverse-chronological, complete inventory of all in-network content that was available to their For You algorithm. For each participant we collect 4,130 inventory posts on average and identify the subset of which that appear in that user's FYP. Despite the fact that we collect in-network inventory posts from a week or more prior to data collection, we observe recency effects for algorithmic amplification, whereby only recent posts are eligible for amplification. Therefore, we subset to all in-network inventory posts that appear in 6-hour time blocks where we observed nonzero amplification i.e. at least one post that appears in their For You feed, resulting in a dataset of 2,952,899 inventory posts across participants.

Our pre-registration can be found at https://aspredicted.org/cg9n-rpqc.pdf. Our primary analysis involves a principal component analysis of the 19 factors. We observe that 4 factors explain 90.37% of variation. We observe that PC1 describes the self/other highest order level of the Schwartz framework with the highest positive loadings on the values of caring (0.361), universal concern (0.354), dependability (0.331), humility (0.274) and tolerance (0.239), and the largest negative loadings on the values of hedonism (-0.3765), self-directed action (-0.264), self-directed thought (-0.2618), stimulation (-0.2526), and achievement (-0.149) in the rotation matrix. PC2 describes the higher level value of conservation in contrast to the self/other highest order level, with the highest positive loadings similar to PC1 on the values of caring (0.3539), dependability (0.305), nature (0.2644), hedonism (0.264) and universal concern (0.25924), but having the largest negative loadings on the conservation values of rule conformity (-0.3691), societal security (-0.3339), tradition (-0.2444), interpersonal conformity (-0.200), and dominance (0.008). PC3 also describes the higher level value of conservation, with the highest positive loadings on the values of rule conformity (0.375), societal security (0.366), tradition (0.337), interpersonal conformity (0.284) and self-directed actions (0.276), and the largest negative loadings on values of caring (-0.009), dependability (0.047), universal concern (0.112), nature (0.136), and personal security (0.152). PC4 distinguishes the higher level values of openness to change and self-enhancement, with the highest positive loadings on the values of achievement (0.548), face (0.459), resources (0.360), dominance (0.254) and personal security (0.0822), and the largest negative loadings on values of self-directed thoughts (-0.307), self-directed actions (-0.304), stimulation (-0.227), rule-conformity (-0.126), and societal security (0.118).

Our primary analysis involves regressing amplification of a post for a person on the principal components of that post for that person with a mixed-effects logistic regression model. The negative statistically significant coefficient on PC1 ($\beta = -0.0051, z = -5.333, p < 0.001$) thus indicates positive amplification of values related to the self, while the negative statistically significant coefficient on PC2 ($\beta = -0.0279, z = -14.964, p < 0.001$) indicates positive amplification of the higher order value of conservation. The positive statistically significant coefficient on PC3 ($\beta = 0.03080, z = 10.470, p < 0.001$) further indicates positive amplification of the higher order value of conservation. The negative statistically significant coefficient on PC4 ($\beta = -0.0147, z = -4.955, p < 0.001$) indicates positive amplification of the higher order value of openness to change.

To understand how these overall β from the principal component analysis map onto the low-level values, we can multiply the rotation matrix describing the loadings above by the β coefficients to reconstruct individual β's for low-level values. Using this approach, we identify a positive amplification of rule conformity ($\beta = 0.02286$), societal security ($\beta = 0.02144$), tradition ($\beta = 0.01728$), interpersonal conformity ($\beta = 0.01449$), self-directed thoughts ($\beta = 0.00833$), self-directed action ($\beta = 0.00833$), stimulation ($\beta = 0.00564$), personal security ($\beta = 0.00312$), dominance ($\beta = 0.00194$), hedonism ($\beta = 0.00160$). We observe a negative amplification of caring ($\beta = -0.01280$), dependability ($\beta = -0.00924$), universal concern ($\beta = -0.00561$), preservation of nature ($\beta = -0.00293$), achievement ($\beta = -0.00253$), tolerance ($\beta = -0.00210$), humility ($\beta = -0.00042$), face ($\beta = -0.00023$), resources ($\beta = -0.00010$).

An additional primary analysis involves regressing amplification of a post for a person on the highest level value clusters of that post (self/other) for that person with a mixed-effects logistic regression model. We observe more amplification of individualistic values ($\beta = 0.023$) than collectivist values ($\beta = 0.002$, p < 0.001).

An additional primary analysis involves regressing amplification of a post for a person on the higher level value clusters of that post (self transcendence, self-enhancement, conservation and openness to change) for that person with a mixed-effects logistic regression model. With this specification, we found a negative statistically significant coefficient on self-transcendence ($\beta = -0.0541, z = -12.638, p < 0.001$), a negative statistically significant coefficient on self-enhancement ($\beta = -0.0361, z = -5.196, p < 0.001$), a positive statistically significant coefficient on conservation ($\beta = 0.1331, z = 18.970, p < 0.001$), and a positive statistically significant coefficient on openness to change ($\beta = 0.0343, z = 6.351, p < 0.001$).

A complementary approach to our primary analysis involves regressing amplification of a post for a person on all of the low level values of that post for that person with a mixed-effects logistic regression model. Using this approach, we identify the significant positive amplification of self-directed thoughts ($\beta = 0.1056, p < 0.001$), interpersonal conformity ($\beta = 0.046, p < 0.001$), face ($\beta = 0.04172, p < 0.001$), societal security ($\beta = 0.0309, p = 0.0134$), resources ($\beta = 0.0387, p = 0.0155$), significant negative amplification of dominance ($\beta = -0.0827, p < 0.001$), self-directed action ($\beta = -0.0510, p = 0.0015$), tolerance

($\beta = -0.0263, p = 0.0089$), hedonism ($\beta = -0.01871, p = 0.029888$), and no effect on achievement ($\beta = -0.00793, p = 0.4495$), stimulation ($\beta = -0.0112, p = 0.40044$), personal security ($\beta = -0.0082, p = 0.3954$), tradition ($\beta = 0.0062319, p = 0.469$), rule conformity ($\beta = 0.000378, p = 0.97241$), humility ($\beta = 0.0132, p = 0.189960$), dependability ($\beta = -0.0213434, p = 0.07902$), caring ($\beta = -0.01282, p = 0.2032$), universal concern ($\beta = 0.0089, p = 0.381$), nature ($\beta = 0.00335, p = 0.4577$)

These results are suggestive but ultimately the collinearity of the low-level model and the nature of PCA make direct low-level value analysis hard to interpret. In order to present maximally interpretable and precise measurements of low-level value amplification, we also present a modification of the higher level value analysis presented above that, for each of the 19, values, includes a low-level value marginal for each of the 19 values separately. In particular, for a given low-level value such as stimulation, we regress the binary indicator for post-level amplification or engagement against the extent to which that post expressed the high-level values of self-transcendence, openness to change, self-enhancement and conservation, and the marginal from subtracting stimulation from openness to change. This process is then repeated 19 times for each low-level value. Then to compute the overall coefficient and significance of the low level value, the coefficient on the marginal (stimulation-openness to change) is added to the coefficient on openness to change. This specification avoids problems of co-linearity while allowing us to identify the unique contribution of low-level values relative to each other.

Using this approach, we identify the significant positive amplification of interpersonal conformity ($\beta = 0.151, p < 0.001$), societal security ($\beta = 0.133, p < 0.001$), rule conformity ($\beta = 0.130, p < 0.001$), tradition ($\beta = 0.123, p < 0.001$), personal security ($\beta = 0.121, p < 0.001$), self-directed thoughts ($\beta = 0.118, p < 0.001$), face ($\beta = 0.072, p < 0.001$), self-directed action ($\beta = 0.0544, p < 0.001$), humility ($\beta = 0.0468, p < 0.001$), significant negative amplification of dominance ($\beta = -0.087, p < 0.001$), dependability ($\beta = -0.056, p < 0.001$), caring ($\beta = -0.051, p < 0.001$), tolerance ($\beta = -0.047, p < 0.001$), universal concern ($\beta = -0.0379, p < 0.001$), hedonism ($\beta = -0.0355, p < 0.001$), preservation of nature ($\beta = -0.0265, p < 0.001$), and achievement ($\beta = -0.0227, p < 0.001$), and no effect on resources ($\beta = -0.000, p = 0.608$) and stimulation ($\beta = -0.0166, p = 0.183$).

These results largely align with the amplification results presented in the main paper.

We note that we focus our inquiry on *the basic values* associated with algorithmic amplification, but future work can use this same method to similarly evaluate these algorithms for political content, misinformation, or other social constructs.

4. Personal values and value alignment

To measure individual values, we used the refined 57-item Portrait Values Questionnaire (PVQ) (5) to compute centered value scores for each participant for each of the 19 values (6). Following the PVQ formulation, we take the average of the three sub-items for each of the 19 values and normalize within participant by subtracting each participants overall average value.

We measure value alignment using the average Spearman rank correlation between each individual's personal values and the frequency of values in the user's in-network inventory, the value-level regression coefficients estimated for amplification, and the value-level regression coefficients estimated for engagement. This framework requires non-missing entries for all 19 low-level values, and we note that our our primary pre-registered value alignment analysis uses n=625/715 entries that have complete non-missing value profiles.

We chose Spearman rank correlation for several reasons. For one, a correlation metric allows us to compare the *relative* expression across all 19 low-level Schwartz values, which follows the relational nature of the value system itself. Further, a correlation computation across values is inherently scale-invariant, allowing us to account for individual level differences in how the scale is used. This also allows us to compare individual values to several different value structures (algorithmic amplification vs inventory distribution vs values associated with engagement) all on the same scale. We chose Spearman rank correlation because the rank ordering captures the ordinal prioritization of values consistent with the underlying theory of basic human values. In addition, a correlation metric handles the ordinal nature of the value annotations, which exist on a 7-point Likert scale.

A. Results and robustness checks. Our primary analysis uses the amplification coefficients from the PCA model. With those values, we observe overall negative correlation between participants' values and the likelihood of algorithmic amplification in a post ($\bar{\rho} = -0.150$, 95% CI=$[-0.169, -0.132]$), which suggests that the algorithm is actually amplifying content that is *misaligned* with users' values. We observe heterogeneity by political identity: Democrats' amplification ($\bar{\rho} = -0.22917$) is more misaligned with their values than Republicans' ($\bar{\rho} = -0.041$, $p < 0.001$).

We also explore the extent to which the main results are robust to different approaches to measuring values. For both of the alternative approaches (marginal and collinear, below), we find qualitatively identical results as the primary analysis).

An alternative approach to measuring values is the marginal method (see Section 3). With those values, we observe overall negative correlation between participants' values and the likelihood of algorithmic amplification in a post ($\bar{\rho} = -0.0569$, 95% CI=$[-0.075, -0.038]$), which suggests that the algorithm is actually amplifying content that is *misaligned* with users' values. We similarly observe heterogeneity by political identity: Democrats' amplification ($\bar{\rho} = -0.1229$) is more misaligned with their values than Republicans' ($\bar{\rho} = 0.035$, $p < 0.001$).

A final alternative approach to measuring values is the collinear method (see Section 3). With those values, we observe overall negative correlation between participants' values and the likelihood of algorithmic amplification in a post ($\bar{\rho} = -0.06522$, 95% CI=$[-0.080, -0.0503]$), which suggests that the algorithm is actually amplifying content that is *misaligned* with users' values. We similarly observe heterogeneity by political identity: Democrats' amplification ($\bar{\rho} = -0.097$) is more misaligned with their values than Republicans' ($\bar{\rho} = -0.020838$, $p < 0.001$).

B. Variation by political posts. We used a political classifier to identify which posts in our dataset are political in nature. The classifier is a distilled language model from fine-tuning a RoBERTa model to identify political posts, including mentions of officials and activists, social issues, news, and current events, based on an annotated training set (7). Within our amplification dataset, 37.29% are political in nature, and we repeat our amplification and alignment analyses for political and non-political content separately.

For political content, we observe overall negative correlation between participants' values and the likelihood of algorithmic amplification in a post ($\bar{\rho} = -0.056$, 95% CI=$[-0.0752, -0.0373]$), which suggests that the algorithm is actually amplifying content that is *misaligned* with users' values. We similarly observe heterogeneity by political identity: Democrats' amplification ($\bar{\rho} = -0.0989$) is more misaligned with their values than Republicans' ($\bar{\rho} = 0.0033$, $p < 0.001$).

For non-political content, we also observe overall negative correlation between participants' values and the likelihood of algorithmic amplification in a post ($\bar{\rho} = -0.088$, 95% CI=$[-0.106, -0.071]$), which suggests that the algorithm is actually amplifying content that is *misaligned* with users' values. We similarly observe heterogeneity by political identity: Democrats' amplification ($\bar{\rho} = -0.143$) is more misaligned with their values than Republicans' ($\bar{\rho} = -0.0125$, $p < 0.001$).

This suggests that our core results (a gap in value alignment for algorithmic amplification) between Republicans and Democrats holds for both political and non-political content and is not driven just by political content.

5. Engagement Analyses

A. Personal Engagement. We use a dataset of engagement events from our participants, collected through browser plugin observation for a period of 24 hours after their initial feed collection. We log engagement events for views, likes, reposts, and replies. In particular, in the 24 hours after feed collection , we observe 31,536 view events from N=1,023 participants and 862 unique engagement events from N=154/1,023 participants that have corresponding value labels. We conduct regression analysis mirroring those for amplification for each engagement action (reply, favorite, repost). In particular, we conduct a regression analysis over posts individuals viewed, predicting a binary indicator of engagement action as a function of the Schwartz values expressed in a post, with random effects for users. We similarly annotate the values in posts using a fine-tuned large language model trained on consensus annotations from a separate sample of 1,079 nationally representative participants (3) and then personalizing at the level of each participant using the personalization procedure outlined above in SI Section 2.

We note however that the engagement analyses presented here comes with some important limitations. They are consistent with the theory of a confrontation effect but do not provide evidence that would conclusively prove mechanism and rule out other explanation for several reasons. For one, when considering individual engagement, we only observed a total of 862 unique engagement events from 154/1,023 participants of which only 6.8% were replies (replying is a sparse form of engagement). This small sample size limits the generalizability of these findings. For another, our pre-registered analysis of partisan differences in value alignment of engagement behavior (e.g. replying) used the personal values of a U.S. representative sample, a population which may have distinct values from the population who 1) self-selected into participating in our feed collection experiment with the Chrome extension, and 2) that subset which replied to posts 24 hours after installing the extension.

B. Global Engagement. Within our amplification dataset, we also collect the overall engagement metrics (number of likes, reposts, and replies) from each post in our participants in-network inventory. We normalize these metrics by log transforming them and conduct a similar regression analysis over inventory posts, predicting log engagement as a function of the Schwartz values expressed in a post, with random effects for users.

It is important to note that when considering global engagement, which is much better powered at detecting a potential confrontation effect and is perhaps more representative of platform dynamics as a whole, there is the concern that we do not observe *who* is engaging with the posts. So while the global engagement results allow us to identify partisan differences in what content attracts engagement, it cannot allow us to say who exactly is interacting with that content. Therefore as a result of these limitations, while the observed partisan differences in value misalignment of X's feed algorithm *are consistent* with a confrontation effect, future work is required in order to demonstrate that a confrontation effect is indeed the causal mechanism underlying the value misalignment of algorithmic amplification (and corresponding partisan difference effects) that we observe.

6. Conceptual replication of alignment analysis

To confirm the robustness of our main results, we conducted a conceptual replication with N=264 recruited from Prolific and quota matched on age, gender and ethnicity. In particular, we collected new data on the values of X/Twitter users after the 2024 election and the change to a Grok-based algorithm change. We then compute the alignment of these users values with the previously measured and pre-registered value amplifications coefficients. This sample had a mean age of 43.53 years, 72.3% white, and 49.6% female and all identified that they use X/Twitter.

For each of these responses, we compute their personal values using the standard PVQ, and then compute their value alignment with the pre-registered metrics from Study 1. Our pre-registration for this replication can be found at `https://aspredicted.org/km4ra5.pdf`.

Overall, we find that algorithmic amplification is value misaligned with these participants ($\bar{\rho} = -0.1041$, 95% CI=$[-0.136, -0.072]$). We found that algorithmic amplification was value aligned for Republicans ($\rho = 0.0372$) and value mis-aligned for Democrats ($\rho = -0.199$) and the difference between the two was statistically significant ($p < 0.001$, pre-registered).

As specified in our pre-registration, we also look at alignment between participant's values and other stages of the social media funnel in the previously pre-registered data, such as base distributions of values in in-network inventory (Following

feed), and posts the original participants engaged with 24 hours after the extension was installed (using the engagement model specification listed in the previous pre-registration).

For in-network inventory, we found that the content of the Following feeds is value aligned with these participants ($\bar{\rho} = 0.2649$, 95% CI=$[0.237, 0.283]$). This was true for both Republicans ($\rho = 0.237$) and for Democrats ($\rho = 0.283$), with no statistically significant difference between the two ($p = 0.117$).

For individual engagement, we found similar patterns to the main analysis.

Individual favoriting was value aligned for both Republicans ($\rho = 0.24266$) and for Democrats ($\rho = 0.2090$), with no statistically significant different between the two ($p = 0.2645$).

For reposting, we found value alignment for both Republicans ($\rho = 0.2064$) and for Democrats ($\rho = 0.3695$), with a statistically significant difference between the two ($p < 0.001$). For replies, we found that replying were value aligned for both Republicans ($\rho = 0.090946$) but value misaligned for Democrats ($\rho = -0.0564$), and the difference between the two was statistically significant ($p < 0.001$, pre-registered).

Following our pre-registration, we also conduct the same analyses but using two different methods for analyzing values - a collinear methods and a marginal method (see pre-registration). The results are largely similar, exhibiting the same qualitative trends with a few minor differences.

For the marginal method, the main result of a partisan difference in alignment for algorithmic amplification holds, as we also find that algorithmic amplification was value aligned for Republicans ($\rho = 0.0816$) and value mis-aligned for Democrats ($\rho = -0.0894$) and a statistically significant difference ($p < 0.001$, pre-registered).

Because the value predictions are highly collinear by construction, the joint specification suffers from multicollinearity, inflating standard errors and leading to unstable coefficient estimates. This results in less precise estimates compared to the other two specifications. For the collinear method, the main result of a partisan difference in alignment for algorithmic amplification is qualitatively similar but not significant ($p = 0.325$): we find that algorithmic amplification was misaligned for Republicans ($\rho = -0.039$) and value mis-aligned for Democrats ($\rho = -0.062$).

Overall, these findings corroborate the continued temporal validity of our results on value alignment, especially in a period post 2024 election and post-algorithm change (Grok), a period when shifting demographics due to platform migration may affect results. Future work should replicate the core value amplification results longitudinally to see how value amplification may or may not change over time (see Section 7).

References

1. Twitter, recap (2023) Accessed: April 3, 2025.
2. X Corp Engineering Team, Twitter's recommendation algorithm (https://blog.x.com/engineering/en_us/topics/open-source/2023/twitter-recommendation-algorithm) (2023).
3. Z Epstein, et al., Whose values? measuring the (subjective) expression of basic human values in social media posts. *Proc. Int. AAAI Conf. on Web Soc. Media* (2026).
4. SH Schwartz, et al., Refining the theory of basic individual values. *J. personality social psychology* **103**, 663 (2012).
5. SH Schwartz, Universals in the content and structure of values: Theoretical advances and empirical tests in 20 countries. *Adv. experimental social psychology/Academic Press.* (1992).
6. SH Schwartz, et al., Extending the cross-cultural validity of the theory of basic human values with a different method of measurement. *J. cross-cultural psychology* **32**, 519–542 (2001).
7. T Piccardi, et al., Reranking partisan animosity in algorithmic social media feeds alters affective polarization. *Science* **390**, eadu5584 (2025).

worker_id

What is your Prolific ID?

Note: Please note that this response should auto-fill with the correct ID

${e://Field/PROLIFIC_PID}

return

Thanks for your participation. The Chrome Extension experienced an unexpected error so please return the submission and message us the code X6hjf

Pilot Consent

DESCRIPTION: You are invited to participate in a research study on social media content. The goal is to understand what kind of values and concepts are embedded in social media content. You will be asked to label social media content according to a set of concepts that we will introduce.

TIME INVOLVEMENT: Your participation will take approximately 45 minutes

total.

RISKS AND BENEFITS: The risks associated with this study are exposure to social media content, which has all the risks associated with reading social media content. Study data will be stored securely, in compliance with Stanford University standards, minimizing the risk of confidentiality breach. The benefits which may reasonably be expected to result from this study are improvements to social media algorithms and design. We cannot and do not guarantee or promise that you will receive any benefits from this study.

PAYMENTS: You will receive $11.25 (~$15/hr prorated based on the estimated study completion time) as payment for your participation.

PARTICIPATION AND WITHDRAWAL: Your participation in this study is completely voluntary and you are free to choose whether to be in it or not. If you choose to be in this study, you may subsequently withdraw from it at any time without penalty or consequences of any kind. The investigator may withdraw you from this research if circumstances arise. If the investigator determines that you are not fulfilling the requirements of the study, he/she may decide to terminate the study early and provide prorated payment.

PARTICIPANT'S RIGHTS: If you have read this form and have decided to participate in this project, please understand your participation is voluntary and you have the right to withdraw your consent or discontinue participation at any time without penalty or loss of benefits to which you are otherwise entitled. The alternative is not to participate. You have the right to refuse to answer particular questions.

CONTACT INFORMATION: Questions: If you have any questions, concerns or complaints about this research, its procedures, risks and benefits, contact the Researcher Investigator, Ziv Epstein (zive@stanford.edu).

If you agree to participate in this research, please click the button to continue.

15

Please enter the number you see in the image (use numerical digits)

Help us keep track of who is paying attention, please select - "Somewhat disagree" in the options below.

- ○ Strongly agree
- ○ Agree
- ○ Somewhat agree
- ○ Neither agree nor disagree
- ○ Somewhat disagree
- ○ Disagree
- ○ Strongly disagree

Instructions

First, you will next be presented with the text of 25 posts from social media. For each post, we are interested in what concepts the post reflects.

value calibration

${lm://Field/1}

${lm://CurrentLoopNumber}/25

To what extent does this post reflect ${lm://Field/2}?

1 - This post does not reflect this concept at all	2 - This post reflects this concept a little bit	3	4	5	6	7 - This post reflects this concept strongly
○	○	○	○	○	○	○

Attention Check 1

When a big news story breaks, people often go online to get up-to-the-minute details on what is going on. We want to know which websites people trust to get this information. We also want to know if people are paying attention to the question. Please ignore the

question and select FoxNews.com and NBC.com as your two answers.

When there is a big news story, which is the one news website you would visit first? (Please choose only one):

- ☐ New York Times website
- ☐ Huffington Post
- ☐ CNN.com
- ☐ FoxNews.com
- ☐ Other ______
- ☐ Yahoo! News
- ☐ NBC.com
- ☐ USA Today website
- ☐ Google News

Demographics

What is your age?

______

What is your gender?

- ○ Male
- ○ Female
- ○ Other

How many years of formal education have you completed?

	0 years	5 years	10 years	15 years	20 years
	0	5	10	15	20
Number of years					

Information about income is very important to understand. Would you please give your best guess?
Please indicate the answer that includes your entire household income in (previous year) before taxes.

- Less than $10,000
- $10,000 - $19,999
- $20,000 - $29,999
- $30,000 - $39,999
- $40,000 - $49,999
- $50,000 - $59,999
- $60,000 - $69,999
- $70,000 - $79,999
- $80,000 - $89,999
- $90,000 - $99,999
- $100,000 - $149,999
- More than $150,000
- Prefer not to answer

Please choose whichever ethnicity that you identify with (you may choose more than one option):

White/Caucasian	Black or African American	American Indian or Alaska Native	Prefer not to answer	Native Hawaiian or Pacific Islander	Asian	Other
○	○	○	○	○	○	○

Are you fluent in English?

- ○ Yes
- ○ No
- ○ Prefer not to answer

Which of the following best describes your political position?

- ○ Democrat
- ○ Republican
- ○ Independent
- ○ [ ] Other (specify)
- ○ Prefer not to answer

Which of the following best describes your political preference?

Strongly Democratic	Democratic	Lean Democratic	Lean Republican	Republican	Strongly Republican
○	○	○	○	○	○

Which, if any, of the following have happened to you, personally, ONLINE?

	Yes, this has happened to me online	No, this has not happened to me online
Been sexually harassed	◯	◯
Been physically threatened	◯	◯
Been stalked	◯	◯
Had someone try to purposefully embarrass you	◯	◯
Been called offensive names	◯	◯
Been harassed for a sustained period	◯	◯

How many accounts do you follow on Twitter/X?

Screener 2

We would like to get a sense of your general preferences.

Most modern theories of decision making recognize that decisions do not take place in a vacuum. Individual preferences and knowledge, along with situational variables can greatly impact the decision process. To demonstrate that you've read this much, just go ahead and select both red and green among the alternatives below, no matter what your favorite color is. Yes, ignore the question below and select both of those options.

What is your favorite color?

- ☐ White
- ☐ Black
- ☐ Red
- ☐ Pink
- ☐ Green
- ☐ Blue

Schwartz Values male

Next we will ask you to rate how similar you are to people with different descriptions. This information is very important for us to understand, so please respond carefully and truthfully.

Here we briefly describe different people. Please read each description and think about how much that person is or is not like you. Please indicate how much the person described is like you.

	Not like me at all	Not like me	A little like me	Moderately like me	Like me	Very much like me
It is important to him to have a good time.	○	○	○	○	○	○

	Not like me at all	Not like me	A little like me	Moderately like me	Like me	Very much like me
It is important to him never to think he deserves more than other people.	○	○	○	○	○	○
It is important to him to avoid upsetting other people.	○	○	○	○	○	○
It is important to him that people do what he says they should.	○	○	○	○	○	○
It is important to him to form his views independently.	○	○	○	○	○	○
It is important to him that the weak and vulnerable in society be protected.	○	○	○	○	○	○
It is important to him that his country is secure and stable.	○	○	○	○	○	○

Here we briefly describe different people. Please read each description and think about how much that person is or is not like you. Please indicate how much the person described is like you.

	Not like me at all	Not like me	A little like me	Moderately like me	Like me	Very much like me
It is important to him to take care of people he is close to.	○	○	○	○	○	○

	Not like me at all	Not like me	A little like me	Moderately like me	Like me	Very much like me
It is important to him to have the power that money can bring.	○	○	○	○	○	○
It is important to him to be tolerant toward all kinds of people and groups.	○	○	○	○	○	○
It is important to him to avoid disease and protect his health.	○	○	○	○	○	○
It is important to him that no one should ever shame him.	○	○	○	○	○	○
It is important to him always to look for different things to do.	○	○	○	○	○	○
It is important to him to care for nature.	○	○	○	○	○	○

Here we briefly describe different people. Please read each description and think about how much that person is or is not like you. Please indicate how much the person described is like you.

	Not like me at all	Not like me	A little like me	Moderately like me	Like me	Very much like me
It is important to him to be wealthy.	○	○	○	○	○	○
It is important to him to have ambitions in life.	○	○	○	○	○	○

	Not like me at all	Not like me	A little like me	Moderately like me	Like me	Very much like me
It is important to him never to violate rules or regulations.	○	○	○	○	○	○
It is important to him to take part in activities to defend nature.	○	○	○	○	○	○
It is important to him that people he knows have full confidence in him.	○	○	○	○	○	○
It is important to him to maintain traditional values and ways of thinking.	○	○	○	○	○	○
It is important to him to make his own decisions about his life.	○	○	○	○	○	○

Here we briefly describe different people. Please read each description and think about how much that person is or is not like you. Please indicate how much the person described is like you.

	Not like me at all	Not like me	A little like me	Moderately like me	Like me	Very much like me
It is important to him to take risks that make life exciting.	○	○	○	○	○	○

	Not like me at all	Not like me	A little like me	Moderately like me	Like me	Very much like me
It is important to him never to annoy anyone.	◯	◯	◯	◯	◯	◯
It is important to him to protect his public image.	◯	◯	◯	◯	◯	◯
It is important to him to be personally safe and secure.	◯	◯	◯	◯	◯	◯
It is important to him to be a dependable and trustworthy friend.	◯	◯	◯	◯	◯	◯
It is important to him to develop his own opinions.	◯	◯	◯	◯	◯	◯
It is important to him to help the people dear to him.	◯	◯	◯	◯	◯	◯

Here we briefly describe different people. Please read each description and think about how much that person is or is not like you. Please indicate how much the person described is like you.

	Not like me at all	Not like me	A little like me	Moderately like me	Like me	Very much like me
It is important to him to have a strong state that can defend its citizens.	◯	◯	◯	◯	◯	◯

	Not like me at all	Not like me	A little like me	Moderately like me	Like me	Very much like me
It is important to him to have the power to make people do what he wants.	○	○	○	○	○	○
It is important to him to follow his family's customs or the customs of a religion.	○	○	○	○	○	○
It is important to him to listen to and understand people who are different from him.	○	○	○	○	○	○
It is important to him to be very successful.	○	○	○	○	○	○
It is important to him to plan his activities independently.	○	○	○	○	○	○
It is important to him to follow rules even when no-one is watching.	○	○	○	○	○	○

Here we briefly describe different people. Please read each description and think about how much that person is or is not like you. Please indicate how much the person described is like you.

	Not like me at all	Not like me	A little like me	Moderately like me	Like me	Very much like me
It is important to him to enjoy life's pleasures.	○	○	○	○	○	○
It is important to him that every person in the world have equal opportunities in life.	○	○	○	○	○	○
It is important to him to honor the traditional practices of his culture.	○	○	○	○	○	○
It is important to him to be the one who tells others what to do.	○	○	○	○	○	○
It is important to him to obey all the laws.	○	○	○	○	○	○
It is important to him to be humble.	○	○	○	○	○	○
It is important to him to figure things out himself.	○	○	○	○	○	○

Here we briefly describe different people. Please read each description and think about how much that person is or is not like you. Please indicate how much the person described is like you.

	Not like me at all	Not like me	A little like me	Moderately like me	Like me	Very much like me
It is important to him to concern himself with every need of his dear ones.	○	○	○	○	○	○
It is important to him to take advantage of every opportunity to have fun.	○	○	○	○	○	○
It is important to him to own expensive things that show his wealth	○	○	○	○	○	○
It is important to him never to be humiliated.	○	○	○	○	○	○
It is important to him to protect the natural environment from destruction or pollution.	○	○	○	○	○	○
It is important to him that people recognize what he achieves.	○	○	○	○	○	○
It is important to him to have all sorts of new experiences.	○	○	○	○	○	○

Here we briefly describe different people. Please read each description and think about how much that person is or is not like you. Please indicate how much the person described is like you.

	Not like me at all	Not like me	A little like me	Moderately like me	Like me	Very much like me
It is important to him to be satisfied with what he has and not ask for more.	○	○	○	○	○	○
It is important to him never to make other people angry.	○	○	○	○	○	○
It is important to him that everyone be treated justly, even people he doesn't know.	○	○	○	○	○	○
It is important to him to avoid anything dangerous.	○	○	○	○	○	○
It is important to him that his country protect itself against all threats.	○	○	○	○	○	○
It is important to him that all his friends and family can rely on him completely.	○	○	○	○	○	○
It is important to him to be free to choose what he does by himself.	○	○	○	○	○	○
It is important to him to accept people even when he disagrees with them.	○	○	○	○	○	○

Schwartz Values female

Next we will ask you to rate how similar you are to people with different descriptions. This information is very important for us to understand, so please respond carefully and truthfully.

Here we briefly describe different people. Please read each description and think about how much that person is or is not like you. Please indicate how much the person described is like you.

	Not like me at all	Not like me	A little like me	Moderately like me	Like me	Very much like me
It is important to her to avoid upsetting other people.	○	○	○	○	○	○
It is important to her that her country is secure and stable.	○	○	○	○	○	○
It is important to her to have a good time.	○	○	○	○	○	○
It is important to her never to think she deserves more than other people.	○	○	○	○	○	○
It is important to her that the weak and vulnerable in society be protected.	○	○	○	○	○	○
It is important to her that people do what she says they should.	○	○	○	○	○	○

	Not like me at all	Not like me	A little like me	Moderately like me	Like me	Very much like me
It is important to her to form her views independently.	○	○	○	○	○	○

Here we briefly describe different people. Please read each description and think about how much that person is or is not like you. Please indicate how much the person described is like you.

	Not like me at all	Not like me	A little like me	Moderately like me	Like me	Very much like me
It is important to her to have the power that money can bring.	○	○	○	○	○	○
It is important to her to be tolerant toward all kinds of people and groups.	○	○	○	○	○	○
It is important to her always to look for different things to do.	○	○	○	○	○	○
It is important to her to take care of people she is close to.	○	○	○	○	○	○
It is important to her that no one should ever shame her.	○	○	○	○	○	○
It is important to her to care for nature.	○	○	○	○	○	○

	Not like me at all	Not like me	A little like me	Moderately like me	Like me	Very much like me
It is important to her to avoid disease and protect her health.	○	○	○	○	○	○

Here we briefly describe different people. Please read each description and think about how much that person is or is not like you. Please indicate how much the person described is like you.

	Not like me at all	Not like me	A little like me	Moderately like me	Like me	Very much like me
It is important to her to make her own decisions about her life.	○	○	○	○	○	○
It is important to her to maintain traditional values and ways of thinking.	○	○	○	○	○	○
It is important to her that people she knows have full confidence in her.	○	○	○	○	○	○
It is important to her to take part in activities to defend nature.	○	○	○	○	○	○
It is important to her to have ambitions in life.	○	○	○	○	○	○

	Not like me at all	Not like me	A little like me	Moderately like me	Like me	Very much like me
It is important to her never to violate rules or regulations.	○	○	○	○	○	○
It is important to her to be wealthy.	○	○	○	○	○	○

Here we briefly describe different people. Please read each description and think about how much that person is or is not like you. Please indicate how much the person described is like you.

	Not like me at all	Not like me	A little like me	Moderately like me	Like me	Very much like me
It is important to her to develop her own opinions.	○	○	○	○	○	○
It is important to her to be personally safe and secure.	○	○	○	○	○	○
It is important to her to be a dependable and trustworthy friend.	○	○	○	○	○	○
It is important to her to take risks that make life exciting.	○	○	○	○	○	○
It is important to her never to annoy anyone.	○	○	○	○	○	○
It is important to her to protect her public image.	○	○	○	○	○	○

	Not like me at all	Not like me	A little like me	Moderately like me	Like me	Very much like me
It is important to her to help the people dear to her.	○	○	○	○	○	○

Here we briefly describe different people. Please read each description and think about how much that person is or is not like you. Please indicate how much the person described is like you.

	Not like me at all	Not like me	A little like me	Moderately like me	Like me	Very much like me
It is important to her to plan her activities independently.	○	○	○	○	○	○
It is important to her to have the power to make people do what she wants.	○	○	○	○	○	○
It is important to her to have a strong state that can defend its citizens.	○	○	○	○	○	○
It is important to her to follow rules even when no-one is watching.	○	○	○	○	○	○
It is important to her to listen to and understand people who are different from her.	○	○	○	○	○	○

	Not like me at all	Not like me	A little like me	Moderately like me	Like me	Very much like me
It is important to her to follow her family's customs or the customs of a religion.	○	○	○	○	○	○
It is important to her to be very successful.	○	○	○	○	○	○

Here we briefly describe different people. Please read each description and think about how much that person is or is not like you. Please indicate how much the person described is like you.

	Not like me at all	Not like me	A little like me	Moderately like me	Like me	Very much like me
It is important to her to obey all the laws.	○	○	○	○	○	○
It is important to her to be humble.	○	○	○	○	○	○
It is important to her to be the one who tells others what to do.	○	○	○	○	○	○
It is important to her that every person in the world have equal opportunities in life.	○	○	○	○	○	○
It is important to her to honor the traditional practices of her culture.	○	○	○	○	○	○

	Not like me at all	Not like me	A little like me	Moderately like me	Like me	Very much like me
It is important to her to figure things out herself.	○	○	○	○	○	○
It is important to her to enjoy life's pleasures.	○	○	○	○	○	○

Here we briefly describe different people. Please read each description and think about how much that person is or is not like you. Please indicate how much the person described is like you.

	Not like me at all	Not like me	A little like me	Moderately like me	Like me	Very much like me
It is important to her to have all sorts of new experiences.	○	○	○	○	○	○
It is important to her that people recognize what she achieves.	○	○	○	○	○	○
It is important to her to protect the natural environment from destruction or pollution.	○	○	○	○	○	○
It is important to her to take advantage of every opportunity to have fun.	○	○	○	○	○	○

	Not like me at all	Not like me	A little like me	Moderately like me	Like me	Very much like me
It is important to her to concern herself with every need of her dear ones.	◯	◯	◯	◯	◯	◯
It is important to her to own expensive things that show her wealth	◯	◯	◯	◯	◯	◯
It is important to her never to be humiliated.	◯	◯	◯	◯	◯	◯

Here we briefly describe different people. Please read each description and think about how much that person is or is not like you. Please indicate how much the person described is like you.

	Not like me at all	Not like me	A little like me	Moderately like me	Like me	Very much like me
It is important to her that everyone be treated justly, even people she doesn't know.	◯	◯	◯	◯	◯	◯
It is important to her never to make other people angry.	◯	◯	◯	◯	◯	◯
It is important to her to avoid anything dangerous.	◯	◯	◯	◯	◯	◯

	Not like me at all	Not like me	A little like me	Moderately like me	Like me	Very much like me
It is important to her that her country protect itself against all threats.	○	○	○	○	○	○
It is important to her to be free to choose what she does by herself.	○	○	○	○	○	○
It is important to her that all her friends and family can rely on her completely.	○	○	○	○	○	○
It is important to her to accept people even when she disagrees with them.	○	○	○	○	○	○
It is important to her to be satisfied with what she has and not ask for more.	○	○	○	○	○	○

Schwartz Values nonbinary

Next we will ask you to rate how similar you are to people with different descriptions. This information is very important for us to understand, so please respond carefully and truthfully.

Here we briefly describe different people. Please read each description and think about how much that person is or is not like you. Please indicate how much the person described is like you.

	Not like me at all	Not like me	A little like me	Moderately like me	Like me	Very much like me
It is important to them that their country is secure and stable.	○	○	○	○	○	○
It is important to them to form their views independently.	○	○	○	○	○	○
It is important to them that the weak and vulnerable in society be protected.	○	○	○	○	○	○
It is important to them never to think they deserves more than other people.	○	○	○	○	○	○
It is important to them to avoid upsetting other people.	○	○	○	○	○	○
It is important to them to have a good time.	○	○	○	○	○	○
It is important to them that people do what they say they should.	○	○	○	○	○	○

Here we briefly describe different people. Please read each description and think about how much that person is or is not like you. Please indicate

how much the person described is like you.

	Not like me at all	Not like me	A little like me	Moderately like me	Like me	Very much like me
It is important to them to be tolerant toward all kinds of people and groups.	○	○	○	○	○	○
It is important to them that no one should ever shame them.	○	○	○	○	○	○
It is important to them to have the power that money can bring.	○	○	○	○	○	○
It is important to them to care for nature.	○	○	○	○	○	○
It is important to them always to look for different things to do.	○	○	○	○	○	○
It is important to them to avoid disease and protect her health.	○	○	○	○	○	○
It is important to them to take care of people they is close to.	○	○	○	○	○	○

Here we briefly describe different people. Please read each description and think about how much that person is or is not like you. Please indicate how much the person described is like you.

	Not like me at all	Not like me	A little like me	Moderately like me	Like me	Very much like me
It is important to them to be wealthy.	○	○	○	○	○	○
It is important to them never to violate rules or regulations.	○	○	○	○	○	○
It is important to them to take part in activities to defend nature.	○	○	○	○	○	○
It is important to them to maintain traditional values and ways of thinking.	○	○	○	○	○	○
It is important to them to make her own decisions about her life.	○	○	○	○	○	○
It is important to them that people she knows have full confidence in her.	○	○	○	○	○	○
It is important to them to have ambitions in life.	○	○	○	○	○	○

Here we briefly describe different people. Please read each description and think about how much that person is or is not like you. Please indicate how much the person described is like you.

	Not like me at all	Not like me	A little like me	Moderately like me	Like me	Very much like me
It is important to them never to annoy anyone.	○	○	○	○	○	○
It is important to them to take risks that make life exciting.	○	○	○	○	○	○
It is important to them to be personally safe and secure.	○	○	○	○	○	○
It is important to them to develop her own opinions.	○	○	○	○	○	○
It is important to them to help the people dear to them.	○	○	○	○	○	○
It is important to them to be a dependable and trustworthy friend.	○	○	○	○	○	○
It is important to them to protect their public image.	○	○	○	○	○	○

Here we briefly describe different people. Please read each description and think about how much that person is or is not like you. Please indicate how much the person described is like you.

	Not like me at all	Not like me	A little like me	Moderately like me	Like me	Very much like me
It is important to them to plan their activities independently.	○	○	○	○	○	○
It is important to them to have the power to make people do what they wants.	○	○	○	○	○	○
It is important to them to follow rules even when no-one is watching.	○	○	○	○	○	○
It is important to them to have a strong state that can defend its citizens.	○	○	○	○	○	○
It is important to them to listen to and understand people who are different from them.	○	○	○	○	○	○
It is important to them to follow their family's customs or the customs of a religion.	○	○	○	○	○	○
It is important to them to be very successful.	○	○	○	○	○	○

Here we briefly describe different people. Please read each description and think about how much that person is or is not like you. Please indicate how much the person described is like you.

	Not like me at all	Not like me	A little like me	Moderately like me	Like me	Very much like me
It is important to them to be humble.	○	○	○	○	○	○
It is important to them that every person in the world have equal opportunities in life.	○	○	○	○	○	○
It is important to them to figure things out themself.	○	○	○	○	○	○
It is important to them to honor the traditional practices of their culture.	○	○	○	○	○	○
It is important to them to obey all the laws.	○	○	○	○	○	○
It is important to them to be the one who tells others what to do.	○	○	○	○	○	○
It is important to them to enjoy life's pleasures.	○	○	○	○	○	○

Here we briefly describe different people. Please read each description and think about how much that person is or is not like you. Please indicate how much the person described is like you.

	Not like me at all	Not like me	A little like me	Moderately like me	Like me	Very much like me
It is important to them never to be humiliated.	○	○	○	○	○	○
It is important to them that people recognize what they achieves.	○	○	○	○	○	○
It is important to them to protect the natural environment from destruction or pollution.	○	○	○	○	○	○
It is important to them to concern themselves with every need of their dear ones.	○	○	○	○	○	○
It is important to them to own expensive things that show their wealth	○	○	○	○	○	○
It is important to them to have all sorts of new experiences.	○	○	○	○	○	○
It is important to them to take advantage of every opportunity to have fun.	○	○	○	○	○	○

Here we briefly describe different people. Please read each description and think about how much that person is or is not like you. Please indicate how much the person described is like you.

	Not like me at all	Not like me	A little like me	Moderately like me	Like me	Very much like me
It is important to them never to make other people angry.	○	○	○	○	○	○
It is important to them to be free to choose what they do by themselves.	○	○	○	○	○	○
It is important to them that everyone be treated justly, even people they doesn't know.	○	○	○	○	○	○
It is important to them to avoid anything dangerous.	○	○	○	○	○	○
It is important to them that all their friends and family can rely on them completely.	○	○	○	○	○	○
It is important to them to accept people even when they disagrees with them.	○	○	○	○	○	○
It is important to them that their country protect itself against all threats.	○	○	○	○	○	○
It is important to them to be satisfied with what they have and not ask for more.	○	○	○	○	○	○

value_perceptions

Twitter/X's algorithm decides which content you see in your "For You" feed. Compared to the posts from the accounts you follow, do you think Twitter's For You algorithm prefers, disprefers, or is neutral on each of the following values?

	Disprefers	Neutral	Prefers
Responsibility: being a reliable and trustworthy member of the ingroup	○	○	○
Caring: devotion to the welfare of ingroup members	○	○	○
Humility: recognizing one’s insignificance in the larger scheme of things	○	○	○
Equality: commitment to equality, justice, and protection for all people	○	○	○
Connection to Nature: care for and connection to the natural environment	○	○	○
Tolerance: acceptance and understanding of those who are different from oneself	○	○	○
Societal security: safety and stability in the wider society	○	○	○

	Disprefers	Neutral	Prefers
Tradition: maintaining and preserving cultural, family, or religious tradition	○	○	○
Lawfulness: compliance with rules, laws, and formal obligations	○	○	○
Respect: avoidance of upsetting or harming other people	○	○	○
Personal security: safety in one's immediate environment	○	○	○
Reputation: security and power through maintaining one's public image and avoiding humiliation	○	○	○
Independent thinking: freedom to cultivate one's own ideas and abilities	○	○	○
Independent actions: freedom to determine one's own actions	○	○	○
Novelty: excitement, novelty, and change	○	○	○
Pleasure: pleasure and sensuous gratification	○	○	○
Power: exercising control over people	○	○	○

	Disprefers	Neutral	Prefers
Wealth: control of material and social resources	○	○	○
Achievement: success according to social standards	○	○	○

random response

Did you respond randomly at any point during the study?

Note: Please be honest! You will get your HIT regardless of your response.

- Yes
- No

Did you search the internet (via Google or otherwise) for any of the news headlines?

Note: Please be honest! You will get your HIT regardless of your response.

- Yes
- No

Did you use AI (e.g. ChatGPT) at any point in the survey?

Note: Please be honest! You will get your HIT regardless of your response.

- Yes
- No

Do you have any comments for us?

Thank you for participating in our study!

Please keep the browser extension installed for 24 hours. After 24 hours, it will provide a link to remove it.

INSTRUCTIONS FOR BROWSER EXTENSION REMOVAL:
Follow the link to the Chrome Store and click "remove from Chrome". Link

If the link does not work:

1. On your computer, open Chrome.
2. At the top right, select More ⋮ and then Extensions and then Manage extensions.
3. On the extension you want to remove, select **Remove**.
4. To confirm, select **Remove**.

Thanks for completing survey! Your completion code is C1O328IX

Powered by Qualtrics
